# From sympathetic storm to systemic inflammation: spatiotemporal dynamics of the brain-lung axis in neurogenic pulmonary edema

**DOI:** 10.3389/fnins.2026.1821390

**Published:** 2026-07-03

**Authors:** Mingxin Zhang, Peng Zhang

**Affiliations:** 1 Department of Neurosurgery, Beijing Tiantan Hospital, Capital Medical University, Beijing, China; 2 Chinese Institutes for Medical Research, Beijing, China

**Keywords:** brain-lung axis, neurogenic pulmonary edema, neuroimmune axis, renin-angiotensin-aldosterone system, sympathetic storm

## Abstract

Neurogenic pulmonary edema (NPE) is a life-threatening complication of acute central nervous system (CNS) injury, characterized by the rapid onset of hypoxemia and pulmonary fluid accumulation in the absence of underlying cardiopulmonary disease. In recent years, emerging integrative frameworks such as the “neuroimmunoaxis” and “brain-lung axis” have provided new perspectives on how CNS injury leads to systemic immune dysregulation and pulmonary dysfunction. However, critical questions remain regarding the interplay between excessive sympathetic activation, immune homeostasis disruption, and lung tissue injury. This narrative review proposes a neurotransmitter-immune-inflammatory model that integrates mechanical, adrenergic, and inflammatory pathways across the spatiotemporal evolution of NPE. We identify four progressive stages involving sympathetic storm initiation due to central autonomic network disinhibition, pulmonary vascular barrier disruption through Piezo channel activation and angiotensin II-norepinephrine synergy, inflammatory amplification from loss of the cholinergic anti-inflammatory reflex, and systemic progression involving gut-lung axis dysregulation. The model generates three testable predictions. Lesions disrupting the nucleus tractus solitarius-ventromedial hypothalamus-intermediolateral column projection should produce more severe NPE. And selective activation of TRPA1^+^ dorsal root ganglion neurons should attenuate sympathetic outflow and pulmonary edema. Enhancing α7 nicotinic acetylcholine receptor signaling should mitigate systemic inflammation. These predictions offer experimental avenues for validating the hijacking hypothesis. Translational implications include stage-specific interventions, early sympathetic blockade, mid-phase anti-inflammatory and neuro-modulatory strategies, and late-stage lung-protective ventilation. This study aims to offer a comprehensive analysis of NPE by exploring its pathological mechanisms—from central sympathetic signaling to peripheral lung damage. Emphasis is placed on examining the interactions between neural signals, neurotransmitters, and immune responses to uncover the spatiotemporal dynamics of NPE. By identifying potential pathways for early diagnosis and targeted therapies, the research seeks to improve disease management and contribute to better clinical outcomes for affected patients.

## 1 Introduction

**FIGURE 1:**
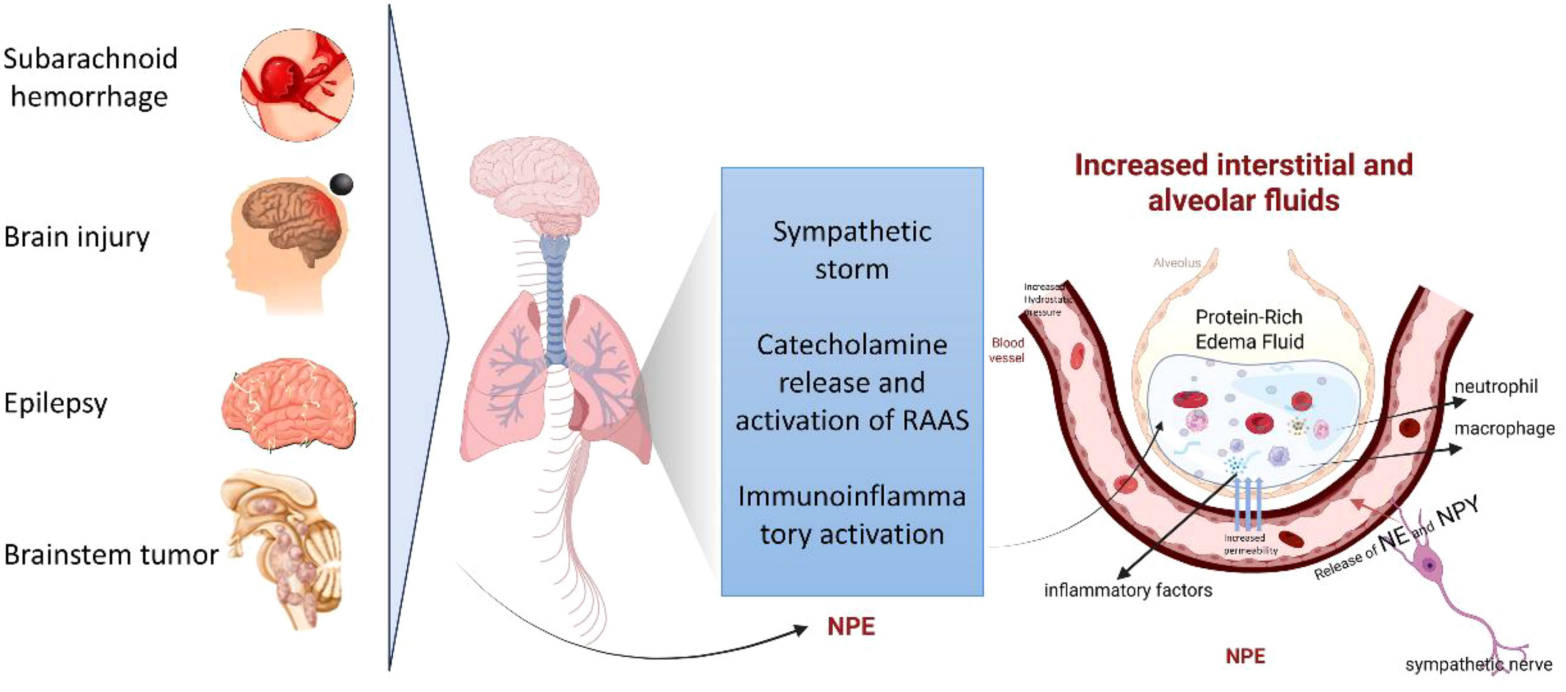
Common causes and pathophysiological mechanisms of neurogenic pulmonary edema. Schematic illustration of the common neurological conditions that trigger neurogenic pulmonary edema and the sympathetic nerve activation pathway involved. Acute central nervous system insults including subarachnoid hemorrhage, traumatic brain injury, epilepsy, and brainstem tumor can stimulate excessive sympathetic nerve outflow. This sympathetic overactivation leads to pulmonary vasoconstriction, increased pulmonary capillary pressure, endothelial disruption, and ultimately pulmonary edema formation. CNS, central nervous system; NPE, neurogenic pulmonary edema, NE, norepinephrine; NPY, neuropeptide Y; RAAS, renin-angiotensin-aldosterone system.

Neurogenic pulmonary edema (NPE) is a severe and often life-threatening complication that arises following acute injury to the central nervous system (CNS), such as subarachnoid hemorrhage, traumatic brain injury, or stroke. It is characterized by the rapid onset of acute hypoxemia and radiographic evidence of pulmonary edema, typically in the absence of primary cardiac or pulmonary pathology in Figure 1 (Finsterer, 2019; Matthay et al., 2012; Zhao J. et al., 2020). The lack of specific biomarkers and the overlap in clinical and radiographic features with conditions like acute respiratory distress syndrome (ARDS) or cardiogenic pulmonary edema make it particularly difficult to identify and diagnose NPE early on. As a result, there are high rates of underdiagnosis and misdiagnosis in clinical settings (Busl and Bleck, 2015; Davison et al., 2012b; Lozada-Martinez et al., 2021; Zhao J. et al., 2020; Ziaka and Exadaktylos, 2021). This could be one of the significant factors contributing to the different reported incidence of NPE from 2% to 42.9% (Kimura et al., 2020; Lozada-Martinez et al., 2021; Nastasovic et al., 2017; Wang L. et al., 2024). Furthermore, mortality rates in severe cases of NPE can reach as high as 60%–100%, especially in patients with extensive CNS damage, further complicating the management of these conditions (Finsterer, 2019; Mascia et al., 2007; Ziaka and Exadaktylos, 2021). Consequently, the need for early, accurate diagnosis and prompt, efficient intervention for NPE has emerged as a pressing clinical issue.

Since its first description in the 20th century, numerous studies have proposed potential pathogenic mechanisms for NPE, yet traditional theories have focused largely on specific components, such as catecholamine release and pulmonary vasoconstriction, without fully accounting for the disease’s dynamic complexity (Lu et al., 2020; Maslonka et al., 2022; Simmons et al., 1969b; Theodore and Robin, 1975; Theodore and Robin, 1976). While these models highlighted important hemodynamic and mechanical components, they offered incomplete explanations for the full spectrum of NPE manifestations—particularly the presence of high-protein edema fluid and the contribution of systemic inflammation. Emerging frameworks, such as the “neuroimmune axis” and “brain-lung axis,” offer integrative perspectives by linking CNS injury to systemic immune dysregulation and pulmonary dysfunction (Azzoni et al., 2024; Bajinka et al., 2022; Jin et al., 2024; Kim et al., 2024; Li et al., 2023). Despite these advances, critical questions remain regarding the relationship between excessive sympathetic activation, immune homeostasis disruption, and pulmonary tissue injury. Emerging technologies, including optogenetics, chemogenetics, and single-cell multi-omics, are driving deeper research into this field and enable the deconstruction of the “central-peripheral” dynamic network, facilitating a comprehensive analysis of the pathological process of NPE, but these techniques have rarely been mentioned in the exploration of the pathogenesis of NPE (Bajinka et al., 2022; Su et al., 2024).

A neuroscientific perspective on NPE: Although NPE manifests as a pulmonary condition, its origin lies squarely within the CNS. Therefore, understanding NPE is, at its core, a neuroscientific challenge. This review moves beyond traditional models that treat the brain as a “black box” and instead endeavors to deconstruct the precise neural map—from higher cortical centers to brainstem autonomic nuclei—that initiates and amplifies this lethal systemic response.

Therefore, this review delves into the pathological mechanisms underlying NPE, with particular focus on the interplay between neural signaling, neurotransmitters, and immune responses. This review was conducted as a narrative integrative review. Literature searches were performed in PubMed and Web of Science for articles published from January 1990 to March 2026 using MeSH terms including neurogenic pulmonary edema, sympathetic storm, brain-lung axis, autonomic nervous system, inflammatory reflex, and neuroinflammation. Inclusion criteria were peer-reviewed original research, clinical studies, and reviews addressing NPE pathophysiology. Exclusion criteria were non-English publications and conference abstracts. Data were synthesized thematically according to the spatiotemporal progression of NPE. By integrating these mechanisms, we aim to provide insight into the spatiotemporal dynamics of the disease and identify potential pathways for early diagnosis and targeted therapy. Ultimately, we hope to contribute to the development of more effective management strategies for this devastating condition, paving the way for improved clinical outcomes.

## 2 The pathogenesis of neurogenic pulmonary edema: from central sympathetic storm to peripheral pulmonary injury

Contemporary research has identified two primary mechanisms underlying NPE: excessive activation of the sympathetic nervous system (SNS) and disruption of the pulmonary vascular barrier (Al-Dhahir et al., 2025; Hasegawa et al., 2022; Kalsotra et al., 2007; Sedy et al., 2015; Yang A. et al., 2022; Zhao J. et al., 2020). These two core mechanisms are tightly interconnected through a “central-peripheral signaling chain,” progressing sequentially from initial mechanical changes to inflammation and oxidative stress. Early pathological changes are primarily mechanical in nature, driven by excessive SNS activation. This is characterized by increased pulmonary capillary pressure and altered Starling forces, both of which facilitate fluid leakage (Theodore and Robin, 1975). Furthermore, the sharp rise in vascular pressure leads to endothelial damage, increasing vascular permeability and triggering further injury (Ziaka and Exadaktylos, 2021). As the pulmonary vascular barrier becomes progressively compromised, immune-inflammatory responses within pulmonary tissue are activated, further amplifying lesion progression and worsening pulmonary edema (Guo et al., 2023; Mrozek et al., 2020; Mutoh et al., 2015). Extensive studies support this progressive pathological progression (Macmillan et al., 2002; Smith and Matthay, 1997; Terrence et al., 1981; Victorio et al., 2016). For example, Smith et al. showed in NPE animal models that pulmonary fluid changes from low - to high - protein exudates, indicating increased permeability as the disease progresses (Smith and Matthay, 1997). Similarly, Victorio et al. (2016) observed an absence of inflammatory cell infiltration during the early stages of NPE, suggesting that mechanical factors predominantly drive the initial pathology prior to the onset of inflammation.

Current theoretical models explaining NPE primarily focus on local mechanisms while largely neglecting the complex interplay between central nerve system injury, neurotransmitters, hormones, and the immune system (Table 1; Davison et al., 2012b; Ziaka and Exadaktylos, 2024).

**TABLE 1:** Theories of pathogenesis mechanism of neurogenic pulmonary edema.

Model name	Core concept	Strengths	Limitations
Neuro-cardiac	*CNS injury triggers excessive sympathetic activation, leading to catecholamine release, myocardial damage, and cardiac dysfunction.	Explains pulmonary edema caused by cardiac dysfunction	Fails to explain cases without cardiac dysfunction; neglects increased vascular permeability and edema fluid complexity.
Neuro-hemodynamic	Sympathetic activation acutely elevates systemic and pulmonary arterial pressures, redistributing blood flow to pulmonary circulation and causing pressure-driven pulmonary edema.	Explains part of the reasons for the occurrence of pulmonary edema	Cannot explain high-protein content in alveolar fluid, suggesting vascular permeability; requires integration with other theories.
Blast theory	Excessive sympathetic activation causes high pulmonary capillary pressure and mechanical damage to the alveolar-capillary membrane (“blast effect”).	Explains acute mechanical injury and high-protein fluid	Focuses on mechanical injury, neglecting inflammation and immune responses; fails to explain elevated inflammatory mediators.
Pulmonary venule adrenergic hypersensitivity theory	Enhanced adrenergic sensitivity of pulmonary veins following CNS injury leads to increased vascular permeability and pulmonary edema.	Supported by animal models	Overlooks inflammation and immune dysregulation; lacks exploration of long-term progression and resolution of NPE.
Double-hit theory	Lung injury occurs in two phases: systemic inflammation from brain injury (first hit) followed by secondary insults like mechanical ventilation or infections (second hit).	Explains elevated inflammatory mediator levels and multi-step lung injury process	Neglects excessive sympathetic activation; overlooks individual differences in inflammatory sensitivity.
Triple-hit hypothesis	Extends double-hit theory by adding “third hit,” where gut dysbiosis through the gut-lung axis exacerbates pulmonary inflammation and injury.	Broadens research on CNS injury and distant organ damage	Insufficiently addresses mechanisms of inflammation amplification, progression dynamics, and individual variability in clinical outcomes.

*CNS, central nervous system.

The neuro-cardiac theory posits that CNS injury triggers excessive sympathetic activation, leading to a dramatic release of catecholamines (Connor, 1969). These neurotransmitters exert toxic effects on myocardial cells, causing myocardial damage and cardiac dysfunction (Zaroff et al., 2000). The resulting cardiac dysfunction leads to pulmonary circulation congestion and pulmonary edema (Bahloul et al., 2006). However, this model fails to explain cases of NPE in which cardiac dysfunction or myocardial damage is absent. Furthermore, it overlooks the role of increased vascular permeability and the complex composition of pulmonary edema fluid.

The neuro-hemodynamic model emphasizes that sympathetic activation causes acute elevations in systemic and pulmonary arterial pressures, redistributing blood flow to the pulmonary circulation and ultimately resulting in pressure-driven pulmonary edema (Sarnoff and Sarnoff, 1952). While animal studies support the increase in pulmonary venous pressure, this model cannot adequately explain the high-protein content observed in alveolar fluid, which suggests increased vascular permeability (Maron, 1989). Although the neuro-hemodynamic model provides valuable insights into NPE’s pathophysiology, it requires integration with other theories to fully explain its complex mechanisms.

The blast theory builds upon the neuro-hemodynamic model, proposing that excessive sympathetic activation not only raises pulmonary capillary pressure but also causes mechanical damage to the alveolar-capillary membrane—a phenomenon referred to as the “blast effect” (Koutsoukou et al., 2016; Theodore and Robin, 1976). This damage increases vascular permeability and accounts for the presence of high-protein fluid in pulmonary edema (Koutsoukou et al., 2016; Maron, 1989). However, the blast theory primarily concentrates on acute mechanical injury mechanisms, neglecting the role of inflammation and immune responses in the long-term progression of NPE (Ziaka and Exadaktylos, 2021). It also fails to explain the elevated levels of inflammatory mediators in the blood or cerebrospinal fluid during NPE pathology.

The pulmonary venule adrenergic hypersensitivity theory suggests that the heightened sensitivity of pulmonary veins to adrenergic stimuli is caused by excessive sympathetic activation following acute brain injury (Koutsoukou et al., 2016; McClellan et al., 1989). This hypersensitivity increases vascular permeability, allowing fluid and proteins to escape into alveolar spaces, ultimately leading to pulmonary edema (van der Zee et al., 1980). Although animal studies support this mechanism, the theory overlooks the contributions of other factors, such as inflammation and immune dysregulation. Additionally, it lacks a detailed exploration of the long-term progression and resolution of NPE.

The double-hit theory attributes lung injury following acute brain injury to a biphasic process (Mascia, 2009). The first hit involves the systemic inflammatory response triggered by brain injury, during which pro-inflammatory cytokines (e.g., IL-1β, IL-6, TNF-α) cross the disrupted blood-brain barrier into systemic circulation, increasing pulmonary vascular permeability and creating vulnerability to secondary insults (Ott et al., 1994; Sweeney et al., 2019). The second hit exacerbates this inflammatory response through secondary factors, such as mechanical ventilation, surgeries, or infections, ultimately aggravating pulmonary injury and edema (Slutsky, 1999; Ziaka and Exadaktylos, 2021). Although the double-hit theory explains the elevated levels of inflammatory mediators observed in patients with NPE, it fails to consider the role of excessive sympathetic activation and overlooks individual variability in sensitivity to secondary inflammatory responses.

The triple-hit hypothesis extends the double-hit theory by introducing a “third hit,” in which gut dysbiosis and intestinal dysfunction, mediated through the “gut-lung axis,” exacerbate pulmonary inflammation and injury (Ziaka and Exadaktylos, 2024). This model broadens the scope of research on the interaction between CNS injury and distant organ damage. However, it does not sufficiently address the mechanisms underlying inflammation amplification, the dynamics of disease progression, or the influence of individual variability on clinical outcomes.

Based on the limitations of existing theories and previous experimental findings, we propose the neurotransmitter-immune-inflammatory model to integrate the multi-phase pathophysiological processes underlying NPE. This model highlights the pivotal role of an initial “sympathetic storm,” wherein acute brain injury triggers excessive sympathetic activation. This activation elevates pulmonary capillary pressure, disrupts Starling forces, and causes mechanical endothelial damage accompanied by oxidative stress. These early events establish a foundation for subsequent immune responses. Then, neuroimmune interactions and the dissemination of brain-derived inflammatory mediators through damaged vascular barriers exacerbate localized inflammation in lung tissues. Immune cells infiltrate through compromised endothelial barriers, further intensifying pulmonary injury and fostering the accumulation of high-protein fluid. This pathological fluid accumulation not only worsens lung injury but also propagates inflammatory processes to other organs. Ultimately, the inflammatory response advances through both localized and systemic pathways, leading to multi-organ failure.

While the triple-hit hypothesis proposed by Ziaka and Exadaktylos (2024) elegantly describes the sequential pulmonary insults following acute brain injury, systemic inflammation, secondary hits, and gut-lung axis dysregulation, it does not fully account for the central neural mechanisms that initiate and perpetuate the sympathetic storm, nor does it explain the transition from hemodynamic to inflammatory phases. Our neurotransmitter-immune-inflammatory model explicitly addresses these gaps by deploying the central autonomic network Central Autonomic Network (CAN) as a functional scaffold to explain how diverse neurological lesions converge onto the same pulmonary phenotype through RVLM disinhibition, incorporating the inflammatory reflex as the missing brake whose disruption permits unchecked cytokine release, identifying the GRK/β-arrestin switch as the molecular mechanism underlying the biphasic action of norepinephrine, proposing that NPE represents a pathological hijacking of the Jin body-brain circuit, and delivering a quantitative spatiotemporal map of NPE progression. Thus, while the triple-hit hypothesis describes what happens to the lung, our model explains how the brain drives and fails to restrain this process.

This model integrates the mechanistic cascade from sympathetic overactivation to immune responses (Huang et al., 2025; Levasseur et al., 1993). It also provides a comprehensive framework for understanding peripheral organ damage induced by acute brain injury. Furthermore, it offers a novel perspective for developing precise intervention strategies and individualized treatments in the management of NPE.

### 2.1 How central injury triggers sympathetic storm: an analysis of the roles of the brainstem and advanced neural centers

The CAN framework, originally conceptualized by Benarroch (1993) and subsequently elaborated by Thayer and Lane (2009), provides an integrated hierarchical model comprising cortical involving anterior cingulate cortex, and insula, subcortical involving amygdala and hypothalamus, brainstem involving periaqueductal gray, nucleus tractus solitarius, and rostral ventrolateral medulla, and spinal including intermediolateral column, structures that collectively regulate autonomic outflow. Within this framework, NPE may be more accurately framed not as a simple surge of sympathetic activation but as a syndrome of disinhibition, the loss of tonic inhibitory control over preganglionic sympathetic output. The medullary nucleus tractus solitarius (NTS) serves as a critical node within the CAN that normally restrains sympathetic outflow via baroreflex-mediated excitation of vagal circuits and inhibition of the rostral ventrolateral medulla (RVLM). Foundational lesion studies by Doba and Reis (1973), Talman et al. (1981) demonstrated that NTS injury removes this baroreflex restraint on the RVLM, leading to explosive hypertension, tachycardia, and pulmonary edema, the cardinal features of NPE. Thus, the convergence of anatomically diverse lesions related to cortical, subcortical, brainstem, or spinal, onto the same pulmonary phenotype can be explained by their common effect: disruption of inhibitory inputs to the RVLM, resulting in unopposed sympathetic outflow. This disinhibition framework not only enriches the conceptual depth of NPE pathogenesis but also provides a unifying principle for understanding how distinct neurological insults produce a shared clinical syndrome.

Sympathetic storm is a pathophysiological response characterized by excessive activation of the SNS, often triggered by CNS injury, such as subarachnoid hemorrhage (SAH), traumatic brain injury (TBI), or acute intracranial pressure (ICP) elevation (Kang et al., 2024; Kim et al., 2024; Ziaka and Exadaktylos, 2024). CNS injury is often accompanied by increased intracranial pressure. A sudden and sharp rise in ICP may trigger the Cushing reflex, leading to the classic triad of hypertension, bradycardia, and irregular respiration (Simmons et al., 1969b). This reflex primarily aims to maintain brain perfusion but also induces excessive sympathetic excitation, which may be associated with the development of pulmonary edema (Chen et al., 1973). In the clinical manifestation of NPE disease, the sympathetic nerve also interacts with other organs after excessive. Its activation leads to myocardial inhibition and a vicious cycle is formed through “heart-brain interaction” - myocardial injury leads to low cardiac output and brain hypoperfusion, further aggravating brain injury and sympathetic activation (Goyal and Bharadwaj, 2019). Concurrently, this process intensifies pulmonary circulatory pressure, aggravating pulmonary edema. As ICP rises to a critical level, it can cause deformation of neural tissue. This leads to brainstem distortion or localized ischemia, disrupting the balance between sympathetic and parasympathetic systems and triggering a sympathetic storm (Thompson and Malina, 1959).

Subarachnoid hemorrhage, a typical form of CNS injury, causes a sharp increase in ICP, directly compressing the brainstem and hypothalamus, making it the leading cause of NPE (Fontes et al., 2003; Friedman et al., 2003; Ochiai et al., 2001). After SAH, hemorrhagic products may directly stimulate the sympathetic nerve-related neural structures, specifically the NPE trigger zones, leading to excessive sympathetic activation and subsequently inducing NPE (Sedy et al., 2015).

**FIGURE 2:**
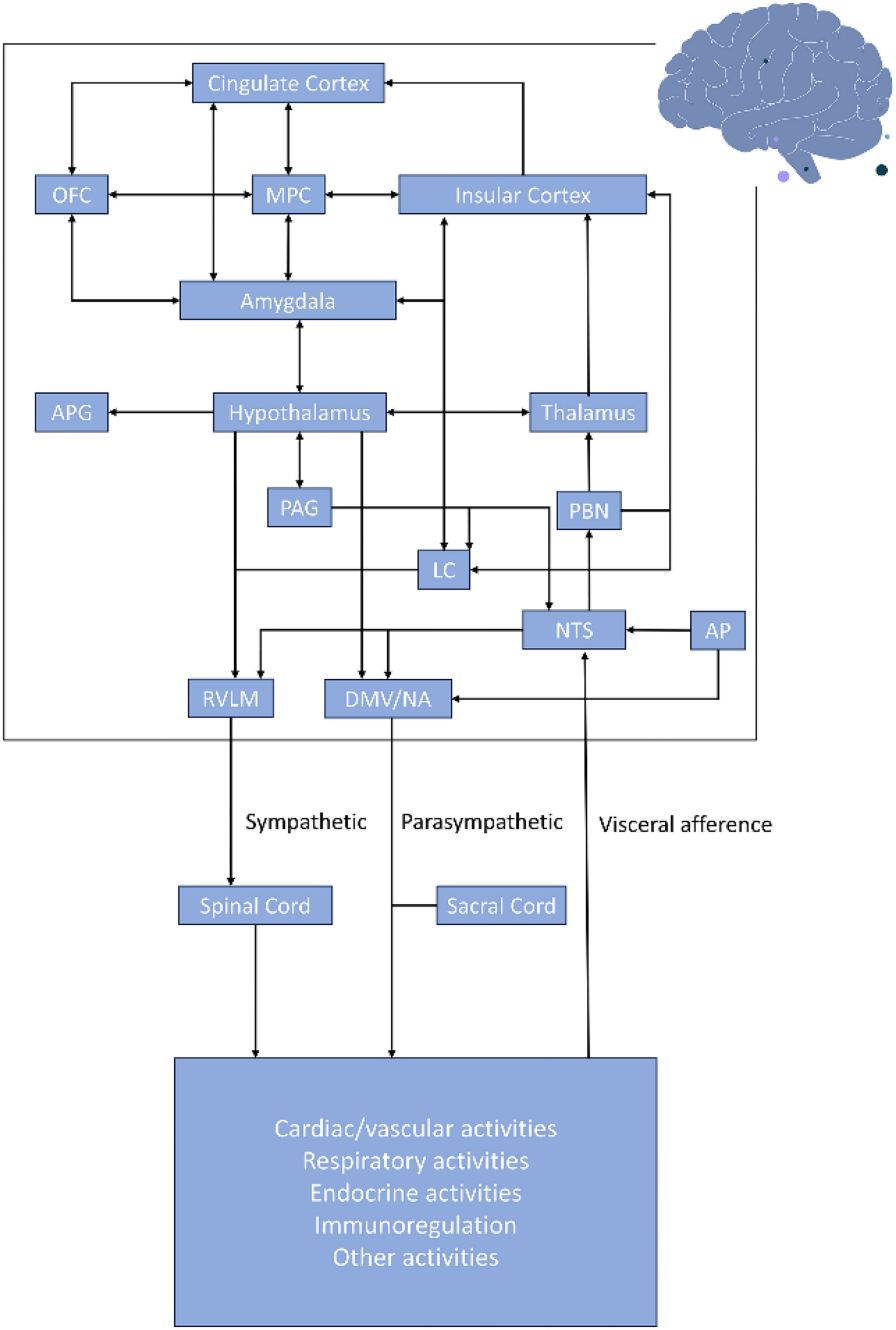
Components of the central autonomic network and its influence on body activities. The central autonomic network comprises cortical, subcortical, brainstem, and spinal structures that collectively regulate autonomic outflow. Cortical and subcortical regions including the cingulate cortex, orbitofrontal cortex, medial prefrontal cortex, insular cortex, amygdala, thalamus, hypothalamus, and anterior pituitary gland integrate sensory and cognitive information. Brainstem nuclei including the periaqueductal gray, parabrachial nucleus, locus coeruleus, nucleus tractus solitarius, area postrema, rostral ventrolateral medulla, dorsal motor nucleus of vagus, and nucleus ambiguus coordinate sympathetic, parasympathetic, and visceral afferent signals projecting to the spinal cord and sacral cord. The CAN ultimately regulates cardiac/vascular activities, respiratory activities, endocrine activities, immunoregulation, and other autonomic functions. AP, area postrema; APG, anterior pituitary gland; DMN, dorsal motor nucleus of vagus; LC, locus coeruleus; MPC, medial prefrontal cortex; NA, nucleus ambiguous; NTS, nucleus tracts solitarius; OFC, orbitofrontal cortex; PAG, periaqueductal gray matter; PBN, parabrachial nucleus; RVLM, rostral ventrolateral medulla.

Current research has revealed that the distribution of NPE trigger zones is relatively extensive, with reports indicating their presence in the cerebral hemispheres, brainstem, and spinal cord. In addition, most of them are the important part of central autonomic network in Figure 2.

In the cerebral cortex, the anterior cingulate cortex (ACC) is closely associated with autonomic nervous system activity. Animal studies have revealed that hyperactivation of the ACC, particularly the subgenual ACC (sgACC/Area 25), can directly or indirectly suppress parasympathetic activity and enhance sympathetic activity through its connections with the brainstem, hypothalamus, and amygdala, thereby leading to autonomic system imbalance (Alexander et al., 2020). This suggests that the ACC may play a potential role in sympathetic hyperactivation observed in NPE. However, its specific role in NPE still needs to be validated by clinical case and mechanism studies.

Insula, as the core of limbic system, has the “lateralization” regulation characteristic of sympathetic-parasympathetic. The right insula leads sympathetic activation (such as increased blood pressure and heart rate), while the left insula enhances parasympathetic activity (Garibotto et al., 2019). However, this lateralization pattern is not absolute, as insular autonomic function varies dynamically according to the antero-posterior subregion stimulated and the experimental context (Oppenheimer and Cechetto, 2016). Clinical stroke studies have yielded conflicting evidence regarding laterality, with some reporting worse outcomes after right insular stroke while others find no significant difference or even adverse events with left insular lesions (Fontes et al., 2024; Vassilopoulou et al., 2020). Clinical case reports have shown that right insular infarction can trigger NPE, suggesting that unilateral insular injury may lead to autonomic imbalance (sympathetic dominance, parasympathetic inhibition) (Tanaka et al., 2023), but the specific molecular mechanisms (such as inflammatory signaling or neurotransmitter release) have not been clarified.

Beyond the cerebral cortex, subcortical structures also play a significant role in autonomic nervous system regulation, and damage to certain subcortical regions has been closely associated with sympathetic overactivation observed in NPE. Previous case reports have indicated that hypothalamic lesions can lead to the development of NPE (Meyer et al., 2009; Yabumoto et al., 1986).

As the central coordinating hub for autonomic regulation, the hypothalamus integrates signals from multiple sources to modulate variables such as heart rate and blood pressure, while also mediating autonomic responses to stress, emotions, and other physiological stimuli (Lamotte et al., 2021). Among its critical components, the paraventricular nucleus (PVN) is particularly important, as it serves as a key component of the hypothalamic-pituitary-adrenal (HPA) axis. Additionally, the PVN contributes to the activation of the sympatho-adrenal-medullary (SAM) axis, leading to catecholamine release, which further enhances SNS activity (Toorie et al., 2016). This cascade of events is generally linked to the effects of catecholamines on neuronal firing rates and receptor expression within the brain, and it is associated with pathological or acute physiological sympathetic hyperactivity and elevated blood pressure (Blair and Ku, 2022; Nagai et al., 2023). Furthermore, the glutamergic neurons of PVN project directly to the extended RVLM and the intermediolateral column (IML) of the spinal cord, activating sympathetic preganglionic neurons, resulting in systemic vasoconstriction and increased cardiac output (Koba et al., 2018). Clinical evidence suggests that hypothalamic injury (e.g., hemorrhage, tumor) can trigger NPE (Yang and Liu, 2020), and the mechanism may be related to overactivation of the PVN-RVLM pathway.

In a clinical case, amygdala injury was found to be associated with the occurrence of NPE (Jacob et al., 2010). The amygdala affects the autonomic nervous system in two ways. The first is the projection of the central amygdala (CeA) into the PVN and brain stem, enhancing sympathetic output (Wang G. et al., 2023). The other is that the amygdaloid-hypothalamic-brainstem circuit releases CRH and norepinephrine (NE) during stress, promoting a “sympathetic storm” (Chaves et al., 2021). Clinical evidence suggests that amygdala damage may induce NPE through the loop disturbances (Kang et al., 2021), but its specific targets still need to be validated.

However, it has been reported that NPE can occur even with midbrain dissociation (blocking supratentorial and subtentorial connections), suggesting that the autonomic nuclei of the brainstem have independent triggering power (Moss et al., 2024). This indicates that there is additional initiation point for NPE located within the brainstem or spinal cord (Chen et al., 1973; Chen, 1995, 2009; Sedy et al., 2015).

The brainstem is a critical structure connecting the brain and spinal cord and is composed of the midbrain, medulla oblongata, and pons (Valenza et al., 2025). Numerous brainstem components are involved in the regulation of autonomic nervous system functions and are closely associated with the pathogenesis of NPE.

Clinical case reports have indicated that patients with midbrain tumors frequently experience significant autonomic dysfunction following surgical intervention (Goh et al., 1999). Additionally, NPE has been observed in some patients with periaqueductal gliomas (Wagle et al., 1990). This phenomenon may be attributed to the disruption of the connections between the periaqueductal gray (PAG) and downstream autonomic centers due to either surgical procedures or tumor-related effects. Furthermore, studies have reported that increased PAG activity can reduce the sensitivity of baroreflex mechanisms, leading to heightened SNS activity, which, in turn, results in increased heart rate and elevated blood pressure (Quadt et al., 2022). These findings underscore the crucial role of the midbrain, particularly the PAG, in autonomic regulation.

Multiple structures within the medulla are closely associated with autonomic nervous activity and the pathogenesis of NPE. The NTS in the medulla is central to regulating autonomic balance. It receives peripheral signals from pressure receptors and chemoreceptors, modulates the activity of the vagal dorsal nucleus and ambiguous nucleus, and inhibits the sympathetic system to maintain the balance between sympathetic and parasympathetic systems (Baranauskas et al., 2023; Guyenet, 2006; Mulkey and Dú Plessis, 2018; Ran et al., 2022; Sévoz-Couche and Brouillard, 2017). In animal experiments, electrical or chemical lesioning of the NTS has been observed to induce explosive hypertension, along with early pathological features of NPE (Doba and Reis, 1973; Pereyra et al., 2023; Talman et al., 1981). This finding underscores the pivotal role of sympathetic-parasympathetic imbalance in the initiation of sympathetic storms. Situated downstream of the NTS, the RVLM modulates pre-sympathetic neurons in the spinal cord via C1-type neurons, serving as a critical regulatory center for sympathetic activity (Farnham et al., 2019). Animal studies have shown that electrical stimulation or excitatory neurotransmitters (e.g., L-glutamate) can significantly increase sympathetic output, triggering fatal pulmonary edema. In contrast, injection of inhibitory neurotransmitters (e.g., GABA) reduces sympathetic overactivation and causes a dose-dependent drop in blood pressure (Ross et al., 1984; Xia et al., 2024).

Except for cerebral hemispheres and brainstem, a potential facilitatory region for NPE also exists in the spinal cord. In a mouse model of NPE induced by spinal cord injury, spinal compression was shown to trigger sympathetic excitation, leading to the onset of sympathetic storm and NPE (He and Nan, 2016; Sedy et al., 2007a,^2012^).

Moreover, studies in the brain-cardiac crossover have found the release of catecholamines due to nerve compression by spinal tumors, leading to abnormalities in the cardiovascular system (Motiejunaite et al., 2021). This suggests that spinal cord compression may lead to overactivity of the SNS, which in turn triggers a pathophysiologic process similar to sympathetic storm, which is related to the mechanism of NPE (Tushara and Neema, 2023). However, severing the spinal cord upstream of the compression site did not result in sympathetic excitation or NPE (Chen, 1995; Sedy et al., 2012). This suggests that although spinal cord injury can induce NPE, it still needs to be activated through the ascending pathway to cause sympathetic overactivation (Sedy et al., 2007b,^2011^). In summary, the core area for NPE development is likely located in the brainstem, especially in the medulla oblongata.

The development of NPE is dependent on the integrity of the sympathetic pathways, as demonstrated in both clinical and animal studies. Soldiers with brain injuries and cervical spinal cord injuries from the Vietnam War did not develop NPE, highlighting that disruption of these pathways can prevent excessive sympathetic activation necessary for NPE (Simmons et al., 1969a; Yang C. et al., 2022; Zhang et al., 2020). Additionally, animal studies have shown that severing the spinal cord at the C7 level effectively prevents NPE induced by brain compression, underscoring the critical role of maintaining sympathetic pathway integrity in NPE development (Chen, 1995).

### 2.2 Pulmonary vascular barrier injury induced by sympathetic storm: from mechanical stress to inflammatory amplification

**FIGURE 3:**
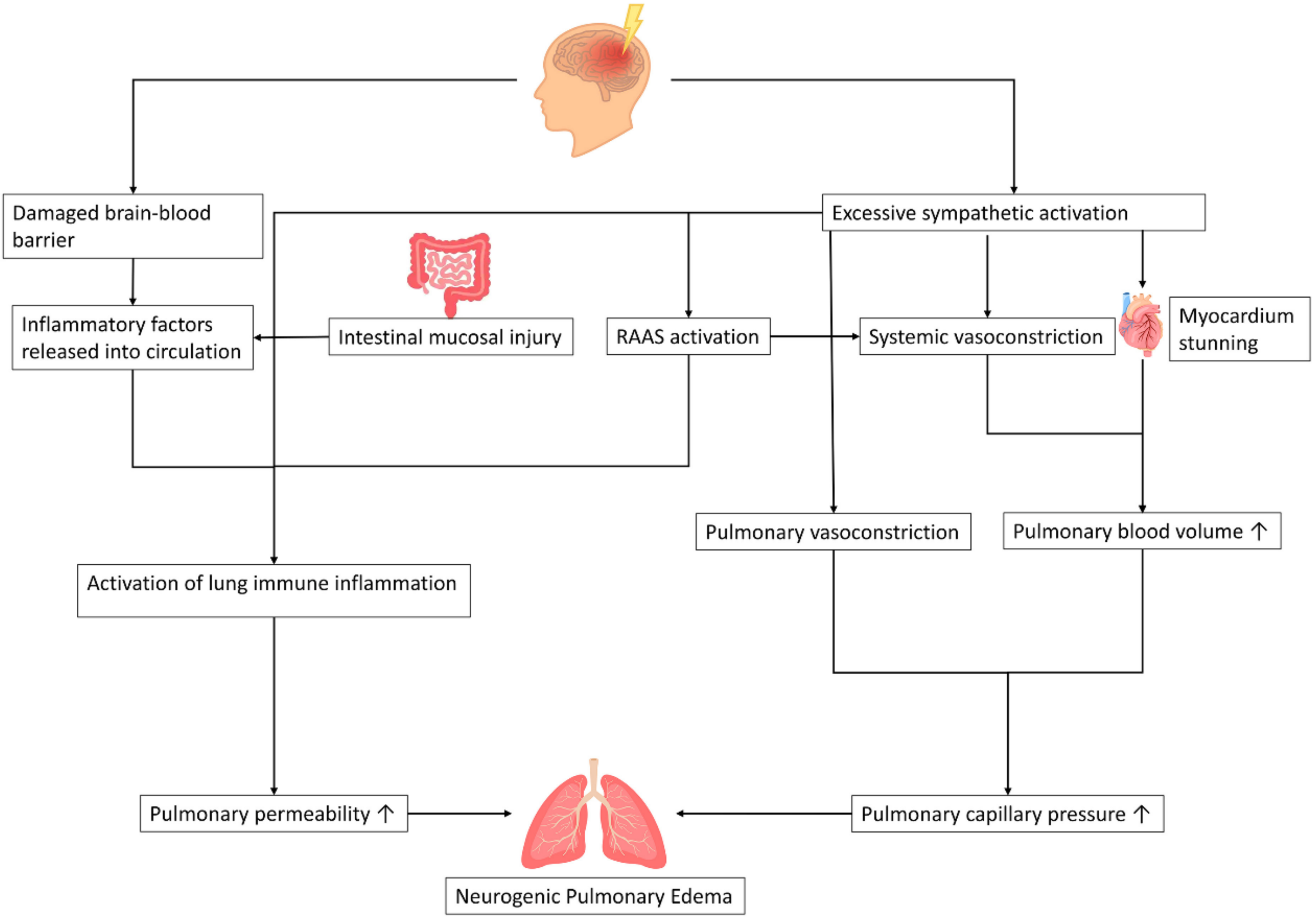
Pathophysiological mechanisms of neurogenic pulmonary edema after acute brain injury. This schematic diagram illustrates the integrated pathways linking acute central nervous system injury to neurogenic pulmonary edema. Acute brain injury leads to three interconnected pathological processes. First, excessive sympathetic activation causes systemic and pulmonary vasoconstriction, increasing pulmonary blood volume, pulmonary capillary pressure, and pulmonary permeability, while also inducing myocardial stunning. Second, disruption of the blood-brain barrier allows inflammatory factors to be released into the systemic circulation, further amplifying pulmonary injury. Third, sympathetic activation stimulates the renin-angiotensin-aldosterone system promoting vasoconstriction and inflammation. Intestinal mucosal injury may also contribute by facilitating the entry of inflammatory mediators. These synergistic mechanisms ultimately converge to produce neurogenic pulmonary edema. RAAS, renin-angiotensin-aldosterone system.

Sympathetic storm often results in excessive release of catecholamines like NE and epinephrine (Ziaka and Exadaktylos, 2021). This response drives systemic vasoconstriction and pulmonary venular constriction, elevating pulmonary circulation pressure and promoting mechanical stress on the pulmonary capillary endothelium (Davison et al., 2012b; Zhao J. et al., 2020; Ziaka and Exadaktylos, 2021). As a result, fluid extravasation into the lung interstitium and alveolar spaces leads to the early development of transvascular exudative pulmonary edema. However, NE-induced mechanical injury is not solely attributable to hemodynamic alterations; myocardial stunning, a condition frequently triggered by excessive catecholamine release, exacerbates pulmonary venous congestion, thereby intensifying hydrostatic stress on the pulmonary vasculature in Figure 3 (Goyal and Bharadwaj, 2019; Kerro et al., 2017; Murthy et al., 2015). Simultaneously, other centrally mediated reflexes, such as Cushing’s reflex, elevate systemic vascular resistance to maintain cerebral perfusion, intensifying damage to pulmonary capillaries (Chen et al., 1973). All mentioned above can significantly worse pulmonary circulation pressure, drive fluid leakage from capillaries into the lung interstitium and result in early “exudative pulmonary edema.”

Mechanical injury plays a crucial role in this progress but remained insufficiently explored until recent advances in understanding mechanosensitive ion channels, particularly the Piezo proteins. The study showed that mechanical damage can trigger an intracellular calcium signaling cascade by activating the Piezo1/2 channel, leading to the breakdown of the pulmonary vascular endothelial barrier and the activation of inflammation (Friedrich et al., 2019; Guo et al., 2021; Sun et al., 2020). In acute lung injury models, high transpulmonary pressure directly activates Piezo1 in alveolar epithelium, promoting calcium influx, NF-κB pathway activation, and increased release of inflammatory factors like IL-6 and TNF-α, thereby enhancing pulmonary vascular permeability (Jiang et al., 2021). In addition, the abnormal activation of Piezo1 was associated with oxidative stress and apoptosis, further amplifying lung tissue damage (Bezerra et al., 2023; Zhang X. et al., 2024). Multiple animal studies have demonstrated the potential of targeting Piezo channels for the treatment of pulmonary edema. In a rat model of ventilator-induced lung injury (VILI), intravenous injection of the Piezo1-specific inhibitor GsMTx4 significantly mitigated VILI-induced lung pathological changes, water content and protein leakage, and induction of systemic inflammatory mediators (Zhang Y. et al., 2021).

While mechanical forces play a significant role in the initial stages of pulmonary injury, the progression of vascular barrier dysfunction is increasingly driven by immune-inflammatory pathways. Historically, it has been proposed that CNS injury induces blood-brain barrier (BBB) disruption, leading to the release of inflammatory signals, such as damage-associated molecular patterns (DAMPs), into the circulation (Bousseau et al., 2023; Jones and Minshall, 2022; Mancia et al., 2020). These factors recruit immune cells (e.g., neutrophils and monocytes) to peripheral organs, including the lungs, leading to augmented endothelial barrier disruption.

Recent advances in understanding the neuro-immune axis provide new insights into how the SNS regulates immune responses (Schiller et al., 2021; Udit et al., 2022). Traditionally, this regulatory mechanism has been associated with immunosuppression, wherein NE binds to β_2_-adrenergic receptors on immune cells, such as macrophages and T cells, activating the cAMP-PKA signaling pathway. This activation inhibits NF-κB phosphorylation and nuclear translocation, thereby suppressing the production of pro-inflammatory cytokines, including IL-6 and TNF-α (Marino and Cosentino, 2013). Demonstrated across in vitro cell lines, primary immune cultures, autoimmune disease models, and human tissue studies (Lorton and Bellinger, 2015). However, some studies on NPE suggest that sympathetic excitation during the development of NPE can trigger immune activation, suggesting a more complex regulatory role for SNS (Belhaj et al., 2022; Naredi et al., 2006; Onohuean et al., 2021; Rostron et al., 2008; Yang A. et al., 2022; Ziaka and Exadaktylos, 2021, 2024).

Recent findings on the body-brain axis highlight Calca^+^ vagal neurons and DBH^+^ CNST neurons as key players in immune modulation via a dynamic balance between pro- and anti-inflammatory states (Jin et al., 2024). This raises the hypothesis that sympathetic nerves may have similar bidirectional immune effects. Accordingly, NE may exhibit a biphasic role. A growing body of evidence supports the hypothesis that the direction of NE signaling, pro-inflammatory versus anti-inflammatory, depends on the receptor subtype engaged and the cellular context. During acute stress or injury, NE is proposed to bind predominantly to α-adrenergic receptors on immune cells, promoting inflammation. While this response aids in anti-infective defense, it may exacerbate capillary damage and vascular leakage in lung injury (Elenkov et al., 2000). In contrast, during chronic stress or prolonged inflammation, NE is hypothesized to signal through β-adrenergic receptors on pericytes and immune cells, inhibiting NF-κB activity and cytokine release, thereby limiting inflammation and promoting tissue protection and repair (Elenkov et al., 2000; Niu et al., 2019; Udit et al., 2022). The molecular switch underlying this biphasic response is thought to involve G protein-coupled receptor kinase (GRK)-mediated phosphorylation of adrenergic receptors, which shifts coupling from G proteins to β-arrestins, thereby altering downstream signaling pathways (Lorton and Bellinger, 2015). This GRK/β-arrestin-dependent signal switching has been implicated in the transition from acute pro-inflammatory to chronic anti-inflammatory state. This proposed mechanism may represent an important homeostatic adaptation, with immune activation in the acute phase counteracting infectious threats and relative immunosuppression in the chronic phase limiting collateral tissue damage and promoting repair (Fajgenbaum and June, 2020). The specific mechanism still needs further confirmation. However, direct evidence for this GRK/β-arrestin switch in the specific context of NPE remains limited, and further experimental validation is required.

Excessive sympathetic activation could also release large amounts of neuropeptide Y (NPY). NPY causes pulmonary vasodilation and increases vascular permeability, worsening pulmonary edema (Barklin et al., 2009; Hirabayashi et al., 1996). Meanwhile, substance P from pulmonary sensory nerves activates local immunity, recruiting neutrophils and further damaging the pulmonary vascular barrier (Yang et al., 2014).

In addition, sympathetic activation also stimulates the renin-angiotensin-aldosterone system (RAAS), forming a synergistic loop with NE (Baffour-Awuah et al., 2024; Benaroua et al., 2025; Jiang et al., 2013; Kalupahana and Moustaid-Moussa, 2024; Reynolds et al., 2020). NE promotes renal renin release, triggering the conversion of angiotensinogen to angiotensin I (Ang I) and subsequently to angiotensin II (Ang II) via ACE in the lungs (Akbari et al., 2022; Bellomo et al., 2020; DiBona and Kopp, 1997; Kumagai et al., 2012; Leisman et al., 2024; Sharp et al., 2018; Silva et al., 2022). Ang II plays a critical role in the pathogenesis of NPE, particularly in amplifying pulmonary vascular damage and immune activation.

Renin-angiotensin-aldosterone system dynamically regulates vascular homeostasis through two antagonistic axes, ACE-Ang II-AT1R and ACE2-Ang (1–7)-Mas (Wu et al., 2022). Dysregulation of the ACE axis leads to a self-perpetuating cycle of injury (Tiwari et al., 2023). Ang II exerts multiple pathological effects by activating angiotensin II receptor type 1 (AT1R). On the one hand, Ang II induces pulmonary vasoconstriction and increases pulmonary vascular resistance, directly damaging the capillary endothelium (El-Arif et al., 2022; Fatima et al., 2021; Forrester et al., 2018; Imai et al., 2005; Schwartz et al., 2023; Shepherd et al., 2023). On the other hand, Ang II activates the NF-κB signaling pathway through AT1R, promoting the release of pro-inflammatory factors (such as IL-6 and TNF-α), recruiting neutrophils and monocytes to infiltrate lung tissue, and further aggravating tissue damage through proteases and other substances (Cantero-Navarro et al., 2021; Chen et al., 2022; Kirça and Yesilkaya, 2023; Ma et al., 2023; Shang et al., 2022; Xie et al., 2022). In addition, Ang II destroys endothelial tight junction proteins (such as claudin-5 and occludin) by generating reactive oxygen species (ROS), thereby increasing vascular permeability and establishing a vicious cycle of inflammation and oxidative stress (Griendling et al., 1994; Liu et al., 2020; Manuneedhi Cholan et al., 2018). These processes collectively lead to the breakdown of the endothelial barrier and persistent lung damage.

When ACE2 activity recovered, Ang II was converted to Ang (1–7). The PI3K/Akt/eNOS pathway is activated through Mas receptors to inhibit NF-κB inflammatory signals and promote vasodilation and endothelial repair. Ang (1–7) can also indirectly reduce oxidative stress by antagonizing AT1R (Xue et al., 2024). Thus, when ACE2/Ang (1–7) axes can counteract the damaging effects of ACE/Ang II, patients may enter a self-healing phase. Therefore, the targeted regulation of ACE and the balance of Ang II/Ang (1–7) may provide preparation for the treatment of pulmonary edema.

Notably, Ang II and NE exhibit synergistic interactions, which amplify immune activation and vascular damage via a positive feedback loop (Clemson et al., 1994; Dhalla et al., 2024; Esler, 2000; Malpas, 2010; Noh et al., 2020; Okusa et al., 2017; Yamamoto et al., 2021). Ang II directly stimulates the adrenal medulla to release catecholamines and enhances NE secretion from sympathetic nerve terminals (Aschrafi et al., 2019; Esler, 2000; Hall et al., 2018; Hardwick et al., 2015). Moreover, Ang II’s effects extend to the central nervous system, where it can act on regions such as the hypothalamus and the RVLM through areas of compromised BBB integrity or regions with naturally thin barriers (Fan et al., 2025; Phillips, 1978; Villapol et al., 2023; Wan et al., 2024; Wu et al., 2024). Also, following CNS injury, the RAAS within the CNS can become activated, leading to increased Ang II production (Ganong, 1984; Villapol et al., 2023). Ang II in the CNS binds to AT1R located on RVLM neurons, where it activates the phospholipase C/protein kinase C (PLC/PKC) signaling pathway, enhancing neuronal excitability and driving sympathetic overactivation (Chen et al., 2010; Zhong et al., 2009). This central mechanism further amplifies sympathetic outflow, elevating NE levels and perpetuating a pathological feed-forward loop between the SNS and RAAS.

This “sympathetic-RAAS feedback loop” drives excessive immune activation, ROS generation, and endothelial permeability, compounding both local pulmonary and systemic inflammatory responses.

### 2.3 The body-brain circuit in neurogenic pulmonary edema

A complete body-brain circuit has recently been elucidated by Jin et al. (2024), who mapped a polysynaptic axis that detects peripheral inflammation and orchestrates counter-regulatory immune responses. This circuit originates from TRPA1^+^ dorsal root ganglion (DRG) sensory neurons innervating peripheral tissues. Upon activation by inflammatory mediators, these sensory afferents project to the nucleus tractus solitarius (NTS) in the medulla oblongata. From the NTS, the signal ascends to the ventromedial hypothalamus (VMH), which then projects to the intermediolateral column (IML) of the spinal cord. These preganglionic fibers innervate the splenic nerve, whose noradrenergic terminals release norepinephrine onto CD4^+^ ChAT^+^ T cells within the spleen. These T cells synthesize acetylcholine, which acts on α7 nicotinic acetylcholine receptors (α7-nAChR) on splenic macrophages, suppressing the release of pro-inflammatory cytokines such as TNF-α, IL-1β, and IL-6. This circuit operates as a closed-loop negative feedback system: peripheral inflammation is sensed by DRG neurons, relayed through the brainstem and hypothalamus, drives sympathetic output to the spleen, and ultimately restrains the same inflammatory response it detected.

In the context of neurogenic pulmonary edema, we propose that this body-brain axis is pathologically hijacked by acute brainstem injury. As detailed in section “2.1 How central injury triggers sympathetic storm: an analysis of the roles of the brainstem and advanced neural centers,” brainstem injury disinhibits the RVLM, unleashing unopposed sympathetic outflow. Consequently, the efferent arc of the Jin circuit becomes saturated with massive catecholamine release, which may overwhelm the α7 nicotinic acetylcholine receptor mediated anti-inflammatory pathway and potentially convert splenic CD4^+^ ChAT^+^ T cells from a regulatory toward a pro-inflammatory phenotype. The net effect is a loss of negative feedback control. Peripheral inflammation can no longer signal the brain to restrain sympathetic output, and the unrestrained sympathetic output drives further inflammation. Neurogenic pulmonary edema may therefore be understood not merely as a syndrome of sympathetic overactivation but as a pathological hijacking of the body-brain feedback loop, in which afferent regulatory signals are disrupted by brainstem injury while the efferent sympathetic arm is disinhibited and amplified.

This hijacking hypothesis integrates the central autonomic network disinhibition framework described, the inflammatory reflex deficit, and the Jin body-brain circuit into a unified mechanistic model. It generates several testable predictions. First, lesions specifically disrupting the nucleus tractus solitarius to ventromedial hypothalamus to intermediolateral column projection should produce more severe neurogenic pulmonary edema. Second, selective activation of TRPA1 positive dorsal root ganglion neurons should attenuate sympathetic outflow and pulmonary edema in neurogenic pulmonary edema models. Third, enhancing α7 nicotinic acetylcholine receptor signaling should mitigate the systemic inflammatory amplification characteristic of neurogenic pulmonary edema. These predictions offer concrete experimental avenues for testing the hijacking hypothesis and for developing novel circuit based therapeutic strategies.

### 2.4 The inflammatory reflex: the missing brake

The classical framework of NPE pathogenesis has focused primarily on efferent sympathetic overactivation, largely overlooking the contribution of afferent and reflex circuits that normally restrain systemic inflammation. The inflammatory reflex, pioneered and subsequently elaborated, represents a hardwired neural circuit that modulates innate immune responses (Pavlov and Tracey, 2005; Tracey, 2002). This reflex arc consists of an afferent limb, vagus nerve sensing peripheral inflammatory signals via its paraganglia and nodose ganglion, and an efferent limb, termed the cholinergic anti-inflammatory pathway, in which acetylcholine released from vagal efferents binds to α7-nAChR on macrophages and other immune cells, suppressing NF-κB nuclear translocation and reducing the synthesis of pro-inflammatory cytokines such as TNF-α, IL-1β, and IL-6 (Wang et al., 2003).

Crucially, the efferent cholinergic anti-inflammatory pathway requires an intact splenic sympathetic nerve and the presence of a specialized population of CD4^+^ T cells that synthesize acetylcholine (ChAT^+^ T cells) within the spleen (Rosas-Ballina et al., 2008, 2011). These T cells relay the vagal signal to splenic macrophages, enabling α7-nAChR-dependent suppression of cytokine release. The vagal-sympathetic-immune interface has been characterized under physiological conditions. However, whether brainstem injury disrupts this multi-synaptic arc specifically in the context of neurogenic pulmonary edema remains to be determined.

As established in sections “2.1 How central injury triggers sympathetic storm: an analysis of the roles of the brainstem and advanced neural centers” and “2.3 The body-brain circuit in neurogenic pulmonary edema,” acute brainstem injury triggers sympathetic storm via RVLM disinhibition and disrupts afferent signaling via NTS involvement. The inflammatory reflex framework adds a critical complementary mechanism: the same brainstem injury compromises the efferent cholinergic anti-inflammatory pathway, removing tonic vagal inhibition of splenic cytokine production (Bustamante et al., 2024). Consequently, the massive catecholamine release saturates splenic CD4^+^ ChAT^+^ T cells with adrenergic input, amplifying systemic inflammation. The combination of reflex failure, afferent brainstem disruption of the vagus-NTS arc, and adrenergic saturation of splenic CD4^+^ ChAT^+^ T cells therefore provides a unified mechanism linking brain injury to uncontrolled pulmonary and systemic inflammation in NPE.

This inflammatory reflex deficit does not replace the disinhibition framework described in the preceding section but rather complements it. While RVLM disinhibition explains the magnitude of sympathetic outflow, inflammatory reflex failure explains why the ensuing inflammatory response escapes endogenous cholinergic restraint, amplifying pulmonary vascular injury and facilitating progression to systemic inflammatory response syndrome and multi-organ failure. Thus, NPE should be understood not only as a syndrome of autonomic imbalance but also as a failure of neuro-immune integration.

### 2.5 Locus coeruleus hyperactivity and glymphatic suppression

The sympathetic-inflammatory cycle after CNS injury is not solely driven by peripheral mechanisms but may also be perpetuated by central noradrenergic pathways that have received limited attention in the NPE literature. The locus coeruleus (LC) is the principal source of NE in the central nervous system (Huang et al., 2012). It projects to virtually all brain regions involved in autonomic regulation, including the hypothalamus, brainstem autonomic nuclei, and the cerebral cortex, and plays critical roles not only in arousal, attention, and stress responses but also in sensory information processing (Huang et al., 2012).

Following acute brain injury, the LC has been reported to exhibit sustained changes in neurotransmitter metabolism. Axonal injury to LC noradrenergic neurons leads to alterations in NE metabolism and synthetic enzyme activity, as demonstrated by changes in NE levels, dopamine-β-hydroxylase, and tyrosine hydroxylase following neurotoxic lesions (Levin, 1983). Importantly, LC hyperactivity has been implicated in driving neuroinflammation. Descending noradrenergic projections from the LC to the spinal cord have been shown to modulate glial cell activation. Selective activation of the LC-spinal cord noradrenergic pathway reduces neuroinflammation by downregulating TNF-α and IL-1β expression, upregulating anti-inflammatory cytokines IL-4 and IL-10, and inhibiting microglia and astrocyte activation (Li J. et al., 2022). These findings suggest that central NE overload can contribute to neuroinflammation through activation of glial cells.

An equally important but frequently overlooked consequence of LC hyperactivity is the suppression of the glymphatic system. The glymphatic system is a recently discovered cerebral waste clearance pathway that facilitates the convective flow of cerebrospinal fluid through the brain parenchyma, clearing metabolic waste products and neurotoxic proteins (Xie et al., 2013). Xie et al. (2013) demonstrated that this system is highly active during sleep but markedly suppressed during wakefulness, with cerebrospinal fluid tracer influx into the brain parenchyma reduced by approximately 95% in the awake state. The interstitial space volume increases by over 60% during sleep, and this state-dependent change is controlled by noradrenergic signaling. In the context of acute brain injury, sustained LC hyperactivity suppresses glymphatic clearance through excessive NE release, leading to the accumulation of damage-associated molecular patterns, pro-inflammatory mediators, and metabolic byproducts within the brain parenchyma (Xie et al., 2013). This accumulation perpetuates neuroinflammation and sustains sympathetic overactivation, potentially contributing to the chronic phase of NPE pathology.

The brainstem noradrenergic system, including the LC, provides direct innervation to key autonomic regulatory centers. The rostral ventrolateral medulla, LC, and paraventricular nucleus provide direct innervation of the intermediolateral column of the spinal cord and are thought to initiate sympathetic responses (Ulrich-Lai and Herman, 2009). Thus, central NE excess may further drive sympathetic outflow through these projections, potentially creating a positive feedback loop that amplifies the peripheral sympathetic storm following CNS injury.

Understanding the role of LC hyperactivity in sustaining neuroinflammation and sympathetic overactivation may provide new insights into the chronic phase of NPE pathology. Therapeutic strategies targeting central noradrenergic tone remain largely unexplored in NPE. Pharmacological modulation of central NE levels, such as with centrally acting agents, represents a potential direction for mitigating sustained neuroinflammation after CNS injury. Future research should investigate whether these strategies can interrupt the sympathetic-inflammatory cycle and improve outcomes in NPE.

### 2.6 Evolution of late-stage NPE: transformation from local to systemic pathological progression

On the basis of early lung barrier injury and localized inflammation, NPE may progressively develop into ARDS, systemic inflammatory response syndrome (SIRS) and further lead to the onset of multi-organ dysfunction syndrome (MODS).

Pulmonary local inflammatory factors (such as IL-6, TNF-α, etc.) and pro-inflammatory molecules released by immune cells progressively enter systemic circulation, profoundly influencing the function of other organs (Ziaka and Exadaktylos, 2021). This systemic influx of endotoxins and pathogens triggers a robust immune response, leading to widespread cytokine release, activation of circulating immune cells, and endothelial dysfunction. Consequently, this heightened inflammatory state can exacerbate multi-organ dysfunction, particularly affecting the lungs, liver, and cardiovascular system. Moreover, the disruption of gut microbiota homeostasis further contributes to immune dysregulation, perpetuating the cycle of inflammation and tissue injury (Ziaka and Exadaktylos, 2024).

Furthermore, damage to the alveolar-capillary barrier causes impaired oxygenation, leaving the patient exposed to sustained hypoxia. Hypoxia, by inducing oxidative stress and inflammatory responses, further contributes to damage to the systemic endothelial barrier (Liao et al., 2023). At this point, the ongoing escalation of inflammation and barrier destruction creates a vicious cycle, and the systemic hypoxia exacerbates the progression of multi-organ dysfunction (Murphy et al., 1993; Quan et al., 2020; Reyes-Pagcatipunan et al., 2024). Eventually, the pathological progression of NPE shifts from early “local pulmonary circulatory disturbance” to “systemic circulatory disturbance,” not only affecting survival rates but also increasing the complexity of clinical management. Thus, early control of the sympathetic storm, stabilization of pulmonary barrier function, and prevention of inflammation spread in the later stages are critical strategies for reducing mortality in NPE patients.

### 2.7 Contradictory evidence and unresolved questions

Although the sympathetic storm hypothesis provides a coherent framework for understanding NPE pathogenesis, several lines of contradictory evidence challenge the completeness of this model and highlight unresolved questions that warrant further investigation.

The catecholamine surge is widely considered a prerequisite for NPE development. However, a case report documented a 6-year-old boy who developed NPE and neurogenic stunned myocardium associated with a brainstem tumor (Moriya et al., 2015). Despite clear clinical and echocardiographic evidence of NPE, his plasma norepinephrine level and urinary norepinephrine level were within normal limits, whereas NPE patients typically exhibit catecholamine levels elevated 10- to 20-fold above normal (Moriya et al., 2015). The authors concluded that pediatric NPE may not be catecholamine-induced, suggesting that alternative mechanisms, such as direct mechanical compression of medullary vasomotor nuclei by hydrocephalus or tumor mass effect, might trigger NPE through pathways independent of systemic catecholamine release (Moriya et al., 2015). This case raises the possibility that NPE pathogenesis may differ across age groups or etiologies, with direct brainstem compression representing a catecholamine-independent mechanism.

If sympathetic overactivation is the primary driver of NPE, β-adrenergic blockade should theoretically be highly effective. Clinical evidence, however, remains inconclusive. While some case reports describe successful treatment of NPE with α-adrenergic antagonists such as phentolamine, the efficacy of β-blockers in NPE has not been systematically established. A randomized controlled trial of esmolol in patients with aneurysmal subarachnoid hemorrhage did not demonstrate significant reduction in NPE incidence or mortality (Davison et al., 2012a). More broadly, the use of β-blockers in acute brain injury remains controversial, with some studies suggesting potential harm due to unopposed a-adrenergic vasoconstriction. The ambiguous outcomes of β-blocker trials suggest that the sympathetic-NPE relationship may be more complex than a simple linear model, possibly involving β-adrenergic receptor polymorphisms, differential receptor sensitivity, or the presence of compensatory mechanisms that bypass adrenergic blockade.

If sympathetic overactivation is necessary for NPE development, then interrupting sympathetic pathways should reliably prevent the condition. Clinical observations, however, reveal important exceptions. Soldiers with concomitant brain and cervical spinal cord injuries during the Vietnam War did not develop NPE, suggesting that intact sympathetic outflow from the brainstem to the periphery is indeed required. Yet, surgical or chemical sympathectomy has not consistently prevented NPE in experimental models or clinical practice. Moreover, NPE has been reported following spinal cord injury below the level of sympathetic outflow, challenging the notion that intact supraspinal sympathetic pathways are absolutely required. These discordant findings suggest that while sympathetic activation is a major contributor, it may not be the sole determinant, and alternative pathways, such as direct neurogenic inflammation or pulmonary vascular α-adrenergic hypersensitivity, may operate independently of central sympathetic outflow.

These contradictory observations do not invalidate the sympathetic storm hypothesis but rather indicate that NPE pathogenesis is likely multifactorial, with the relative contributions of hemodynamic, neurogenic, and inflammatory mechanisms varying across patients, etiologies, and disease stages. The neurotransmitter-immune-inflammatory model proposed here makes several testable predictions. However, certain observations would substantially challenge or refute this framework. If NPE were reliably induced in patients with complete cervical spinal cord transection, eliminating sympathetic outflow from the brainstem to the periphery, this would argue against the necessity of sympathetic storm as the primary driver and would require a major revision of the model. Besides, if selective pharmacological blockade of the renin-angiotensin-aldosterone system completely abolished NPE without any effect on sympathetic outflow, this would suggest that the sympathetic-RAAS synergy is not essential, and the model’s emphasis on sympathetic-RAAS crosstalk would need to be reconsidered. And if enhancing α7 nicotinic acetylcholine receptor signaling in the absence of an intact splenic nerve or CD4^+^ ChAT^+^ T cells still attenuated pulmonary edema, this would challenge the proposed efferent arc of the body-brain circuit and the inflammatory reflex framework. Explicitly identifying these falsifiable predictions strengthens the scientific rigor of the proposed model. Future research should systematically characterize patient subgroups in whom catecholamine-independent mechanisms predominate, conduct adequately powered trials of β-blockers with pharmacodynamic monitoring, and elucidate the conditions under which sympathetic denervation succeeds or fails to prevent NPE.

## 3 Therapeutic interventions for NPE: targeted strategies based on pathophysiological mechanisms

The management of NPE requires a multidisciplinary approach that integrates rapid control of the primary neurological insult with targeted modulation of downstream pathophysiological cascades. The therapeutic framework is stratified into several key domains, supported by preclinical and clinical evidence.

### 3.1 Treatment strategies for sympathetic storm caused by central nervous system injury

#### 3.1.1 Primary disease control and ICP management

Neurogenic pulmonary edema caused by conditions such as acute cerebrovascular disease, traumatic brain injury, and intracranial hemorrhage requires prompt management of the underlying neurologic insult. Treating the primary disease—such as hematoma evacuation, dura repair, or decompressive surgery—is crucial to reducing brainstem distortion, sympathetic overactivation, and subsequent pulmonary complications (Bernhardt et al., 2024; Finsterer, 2019; Lo-Cao et al., 2021).

Lowering ICP is a key priority for alleviating NPE. Mannitol, a commonly used hypertonic agent, reduces ICP by drawing water from brain tissue into the vasculature for excretion via the kidneys (Toung et al., 2005). A review integrates clinical experience and research on the effective use of mannitol, offering insights to optimize therapeutic strategies, enhance patient outcomes, and guide future research (Kim et al., 2023). In addition, adjunctive measures such as short-term hyperventilation and propofol sedation temporarily reduce cerebral metabolic demand and peak ICP. In a retrospective study six patients with NPE were found to be relieved of dyspnea and resumed discharge by reduction of ICP and ventilator-assisted respiration (Lu et al., 2022).

#### 3.1.2 Autonomic nervous system modulation

Sympathetic overdrive, a hallmark of NPE, leads to pulmonary vasoconstriction and endothelial injury, triggering sudden increases in capillary pressure, permeability, and fluid extravasation. α-Adrenergic antagonists (e.g., phentolamine) directly attenuate pulmonary vascular resistance (Xiao-Qun et al., 2014), whereas β1-selective blockers (e.g., esmolol) attenuate cardiac hyperdynamic and myocardial oxygen demand (Peltenburg et al., 2022; Sinagra et al., 2020). In addition, nitroglycerin reduces left heart preload by venodilation, and a randomized controlled trial found nitroglycerin to have a therapeutic effect on sympathetic storm-induced pulmonary edema (Naazia et al., 2023). Dexmedetomidine, a centrally acting α2 agonist, inhibits sympathetic outflow while minimizing respiratory depression, providing an advantage over volatile anesthetics in the intensive care setting (Zhao Y. et al., 2020). In addition to pharmacological sympathetic inhibition, a single preliminary study in 23 rabbits reported a correlation between degeneration of the T3 dorsal root ganglion (DRG) and higher NPE scores, suggesting a potential link between thoracic DRG integrity and NPE severity (Sirinoglu et al., 2024). However, these findings are derived from a small exploratory study with limited sample size and have not been replicated in independent cohorts or larger animal models. Moreover, no clinical data are currently available to support the therapeutic relevance of targeting T3 DRG in human NPE. Therefore, while this observation raises an interesting hypothesis, further rigorous studies, including larger animal experiments and, if warranted, clinical investigations, are required to validate these exploratory findings and assess their potential translational implications.

### 3.2 Treatment strategies for pulmonary vascular injury caused by sympathetic storm

#### 3.2.1 Anti-inflammatory and antioxidant therapy

Sympathetic storms are usually accompanied by hemodynamic instability, which affects pulmonary circulation, which in turn leads to pulmonary vascular injury. In turn, inflammatory mediators (e.g., TNF-α, IL-6) and oxidative stress exacerbate endothelial permeability. Antioxidants such as activators of the Nrf2 signaling pathway have been proposed as potential therapeutic means that can attenuate the damage of oxidative stress on lung tissue (Hamblet et al., 2024; Hu et al., 2024; Zhang Y. et al., 2021). N-acetylcysteine (NAC) scavenges reactive oxygen species and supplements glutathione and stabilizes alveolar capillary membranes (Chen, 2011). And NAC can attenuate oxidative damage, protect lung endothelial cells, and further stabilize lung function (Fu et al., 2024; Kumar et al., 2023; Zhao Y. et al., 2020). Ulinastatin, a serine protease inhibitor, reduces neutrophil elastase activity in animal models, but there is a lack of standardization of human dosing regimens (Horváth et al., 2022). S100A9, secreted primarily by neutrophils and monocytes, is a calcium-binding protein involved in inflammatory responses and innate immune regulation (Naaman et al., 2024; Paramasivam et al., 2024; Sun et al., 2024). It has been found that knockdown of S100A9 attenuates NPE, and that Paquinimod, a specific inhibitor of S100A9, also attenuates NPE, suggesting that modulation of proteins associated with the inflammatory response is one of the directions for the treatment of NPE (Wang G. et al., 2023). In addition, targeted biologics, such as anti-TNF-α drugs, are still being investigated in NPE. Other biological therapies, such as anti-TNF-α antibodies, are under investigation. IFN-β has demonstrated pulmonary immunomodulatory effects in experimental aneurysmal SAH models. In such studies, IFN-β reduced lung pro-inflammatory markers, including TNF-α, showcasing its therapeutic potential (Cobelens et al., 2010). Vitamin D (VitD) has also been researched for its role in immune and inflammatory responses. A prospective study involving 138 children with hand-foot-mouth disease found significantly lower serum 25(OH)D levels in deceased patients compared to survivors. Reduced VitD was associated with higher occurrences of brainstem encephalitis, NPE, and circulatory failure, indicating its possible role in immunity and NPE prevention (Dang et al., 2017).

#### 3.2.2 RAAS pathway inhibition

Sympathetic activation potentiates RAAS-mediated fluid retention and pulmonary hypertension. While ACE inhibitors (e.g., captopril) reduce angiotensin II generation, their use in acute NPE is limited by hypotensive risks (Chen et al., 2014). Angiotensin receptor blockers (ARBs, e.g., losartan) and aldosterone antagonists (e.g., spironolactone), may offer alternative RAAS blockade, though clinical evidence is confined to case series. In the management of acute NPE, the selection of RAAS blockers requires a combination of the patient’s hemodynamic status and potential risks. For example, ARBs and aldosterone antagonists may be more appropriate for use in those patients at higher risk for hypotension (Herrmann et al., 2022). Notably, RAAS inhibitors should be deferred until hemodynamic stability is achieved.

#### 3.2.3 Neuromodulatory approaches

Beyond pharmacological sympathetic blockade, neuromodulatory interventions directly targeting the cholinergic anti-inflammatory pathway have emerged as promising adjunctive strategies for NPE. Vagus nerve stimulation (VNS), particularly non-invasive transauricular VNS, has been shown to activate the efferent cholinergic anti-inflammatory pathway, reducing systemic cytokine levels and attenuating organ injury in preclinical models of sepsis and systemic inflammation (Koopman et al., 2016). Although direct evidence in NPE models remains limited, the mechanistic rationale is compelling, VNS enhances acetylcholine release, which binds to α7-nAChR on immune cells, suppressing NF-κB activation and reducing pro-inflammatory cytokine production. Selective α7-nAChR agonists, such as GTS-21 and encenicline, have demonstrated anti-inflammatory effects in experimental models of acute lung injury and may offer a more targeted approach without the technical challenges of device-based stimulation (Nakamura et al., 2023; Salaga et al., 2016). Local anesthetics and Neuraltherapeutic Medicine have also been proposed as emerging complementary modalities for modulating sympathetic outflow and resetting autonomic balance, though clinical evidence specific to NPE is currently lacking. These neuromodulatory approaches should be considered as adjunctive strategies, with their use guided by hemodynamic status and institutional expertise.

#### 3.2.4 Immunomodulation and barrier protection

Oxidative stress is a key driver of pulmonary vascular injury in NPE. Several antioxidant agents have mechanistic rationale for attenuating the oxidative component of pulmonary vascular injury. N-acetylcysteine (NAC) scavenges reactive oxygen species and replenishes glutathione, thereby stabilizing alveolar capillary membranes and protecting lung endothelial cells (Chen, 2011). High-dose intravenous vitamin C has demonstrated endothelial-protective and hypoxia-inducible factor 1α (HIF-1α)-modulating effects in sepsis-related literature, which may be extrapolated to the oxidative stress observed in NPE. Vitamin D, partially addressed earlier, vitamin E, and alpha-lipoic acid also exhibit antioxidant properties that may reduce endothelial permeability and mitigate lung injury (Dang et al., 2017). These agents should be positioned as adjunctive therapies with preclinical plausibility rather than primary interventions.

A growing body of preclinical evidence supports the use of natural compounds with anti-inflammatory and endothelial barrier-protective properties. Resveratrol, curcumin, quercetin, epigallocatechin-3-gallate (EGCG), and omega-3 fatty acids exert documented effects on NF-κB signaling, NLRP3 inflammasome activation, and endothelial barrier integrity (Jiang et al., 2019; Wang Y. et al., 2024). These compounds have shown promise in reducing cytokine release, attenuating oxidative stress, and preserving vascular barrier function in various models of acute lung injury. However, clinical evidence specific to NPE is lacking, and these agents should be presented as adjunctive strategies with preclinical plausibility, properly graded in evidence level, rather than as primary therapies. Future translational studies are needed to determine their efficacy and safety in the NPE setting.

### 3.3 Late stage NPE management

#### 3.3.1 Mechanical ventilation and the PEEP-ICP dilemma

Mechanical ventilation remains a cornerstone of respiratory support for patients with severe NPE. When respiratory failure develops, timely initiation of intubation and mechanical ventilation effectively improves oxygenation and ventilation (Fletcher and Atkinson, 2003). However, a central clinical dilemma in NPE intensive care is the tension between positive end-expiratory pressure (PEEP) and intracranial pressure (ICP). High levels of PEEP, while beneficial for oxygenation and alveolar recruitment, may impair cerebral venous return and elevate ICP, potentially worsening the primary neurological insult (Humayun et al., 2022). Conversely, low PEEP may fail to maintain adequate oxygenation and promote alveolar collapse.

Current recommendations advocate for a balanced approach. Permissive hypercapnia, within a limited range, may be tolerated to avoid excessive tidal volumes and high intrathoracic pressures. PEEP titration should be guided by both oxygenation goals and ICP monitoring, with most guidelines suggesting PEEP levels of 5–10 cmH_2_O in the absence of refractory intracranial hypertension. For patients with elevated ICP, PEEP should be carefully titrated while maintaining cerebral perfusion pressure (Humayun et al., 2022). Prone positioning has been shown to enhance ventilation-perfusion matching and improve oxygenation in severe hypoxemia, and may be considered in refractory cases, provided that ICP can be closely monitored and controlled (Humayun et al., 2022; Marshall and Nyquist, 2009). The use of prone positioning in NPE requires careful attention to head and neck positioning to avoid further increases in ICP.

#### 3.3.2 Respiratory support and preventative measures

Mechanical ventilation is a critical means of respiratory support for patients with severe disease. When patients develop respiratory failure, timely administration of tracheal intubation mechanical ventilation can effectively improve ventilation and oxygenation (Fletcher and Atkinson, 2003; Frisvold et al., 2023; Ziaka and Exadaktylos, 2021). The patient’s respiratory function is maintained by adjusting the appropriate ventilation mode and parameters, such as tidal volume, respiratory rate, and positive end-expiratory pressure. For severe hypoxemia, prone position is recommended to enhance ventilation-perfusion matching (Fletcher and Atkinson, 2003; Marshall and Nyquist, 2009). In addition, emerging therapies, including endothelial stabilizers (e.g., recombinant angiotensin-converting enzyme 2) and microcirculatory modulators (e.g., iloprost), show preclinical promise but require validation in NPE-specific trials. In high-risk patients, vital signs, including heart rate, blood pressure, respiratory rate, and oxygen saturation, should be closely monitored (Sedý et al., 2009). Once abnormal changes are detected, further examination and treatment should be carried out promptly. For example, when patients experience symptoms such as slowed heart rate, elevated blood pressure, and shortness of breath, they should be alerted to the occurrence of NPE and take appropriate preventive measures. In addition, identifying risk factors such as infection, hypoxemia, and acidosis are also important measures to prevent NPE (Mura et al., 2004).

In terms of fluid management, fluid overload should be avoided, and pulmonary edema should be effectively managed with diuretics such as furosemide (Clark and Soos, 2025; Ergezen et al., 2023; Pandey et al., 2000; Parepalli et al., 2024; Vergouwen and Rinkel, 2023).

## 4 Future direction

The exploration of NPE necessitates a systems-based approach, as the condition arises from complex interactions between neural, immune, and inflammatory processes. Future research should integrate the analysis of spatiotemporal pathological dynamics with advanced techniques such as optogenetics, chemogenetics, and genomics for targeted interventions, emphasizing precision therapy through interdisciplinary collaboration.

### 4.1 Spatiotemporal dynamic mechanism analysis

Neurogenic pulmonary edema progresses through a temporal cascade that can be divided into four distinct stages based on pathophysiological events, time course, and therapeutic opportunities. Understanding these spatiotemporal dynamics provides a theoretical basis for stage-specific interventions and informs the design of future clinical research.

Stage I is sympathetic storm initiation, occurring from minutes to hours after the initial insult. Immediately following acute brain injury, disinhibition of the rostral ventrolateral medulla, or RVLM, due to loss of tonic inhibitory input from the nucleus tractus solitarius, or NTS, triggers a massive surge in sympathetic outflow (Doba and Reis, 1973). This results in explosive release of norepinephrine, also known as NE, and neuropeptide Y, or NPY, driving systemic and pulmonary vasoconstriction. The therapeutic window for this stage is ultra-early, within one to 2 h, with interventions including intracranial pressure control, α-adrenergic antagonists such as phentolamine, and centrally acting α*2* agonists such as dexmedetomidine.

Stage II is the pulmonary vascular barrier disruption, which usually begins within minutes to hours after the initial insult. The sustained sympathetic surge activates the renin-angiotensin-aldosterone system, or RAAS, generating angiotensin II, or Ang II, which synergizes with NE to amplify vasoconstriction and endothelial injury (Friedrich et al., 2019). Concurrently, elevated pulmonary capillary pressure activates mechanosensitive Piezo1 and Piezo2 channels, triggering calcium influx, NF-κB activation, and disruption of endothelial tight junctions. The therapeutic window for this stage is early, approximately 2–6 h, with interventions including ACE inhibitors, angiotensin receptor blockers, and Piezo1 antagonists such as GsMTx4.

Stage III is the inflammatory amplification phase, spanning from 6 to 72 h after the initial insult. Brainstem injury triggers a triad of pathological events, namely disruption of afferent signaling, RVLM disinhibition with sympathetic storm, and loss of the cholinergic anti-inflammatory reflex. The convergence of these mechanisms permits the unchecked release of pro-inflammatory cytokines such as IL-1β, IL-6, and TNF-α from activated immune cells (Wang et al., 2003). The therapeutic window for this stage is intermediate, approximately 6–48 h, during which interventions including α7-nAChR agonists such as GTS-21, vagus nerve stimulation, anti-TNF-α antibodies, and N-acetylcysteine may be considered.

Stage IV is systemic progression, extending beyond 72 h. Persistent sympathetic overactivation leads to locus coeruleus, or LC, hyperactivity, which suppresses glymphatic clearance, allowing accumulation of neurotoxic mediators (Zhu et al., 2025). Concurrently, gut dysbiosis and increased intestinal permeability activate the gut-lung axis, fueling systemic inflammation. This stage culminates in acute respiratory distress syndrome, or ARDS, systemic inflammatory response syndrome, or SIRS, and multi-organ dysfunction syndrome, or MODS. The therapeutic window for this stage is late, beyond 48–72 h, with interventions including lung-protective ventilation with careful PEEP-ICP titration, prone positioning, glycemic control, and supportive care.

Neurogenic pulmonary edema progresses through a temporal cascade, starting with sympathetic overactivation (sympathetic storm), which induces neurotransmitter surges (e.g., NE) that exacerbate lung inflammation, trigger cytokine release (e.g., IL-6, TNF-α), and aggravate lung injury. Although the timing and interaction of these factors remain unclear, future research should focus on mapping the spatiotemporal dynamics from early sympathetic activation to inflammation spread. NPE manifests distinct stages: early, characterized by neuromodulatory imbalance and increased capillary permeability; middle, defined by elevated inflammatory indexes; and late, marked by persistent pulmonary edema, alveolar instability, atrophy, and gas exchange impairment. These stages provide guidance for targeted interventions: in the early stage, sympathetic blockers can regulate neuromodulation and capillary permeability; in the middle stage, anti-inflammatory drugs can address the inflammatory response; and in the late stage, severe cases may require mechanical ventilation, glucocorticoids to reduce inflammation and fibrosis, or antibiotics to prevent infections. In conclusion, understanding the mechanism of temporal and spatial dynamics of NPE helps to understand the pathophysiologic process of the disease, provides a theoretical basis for the development of new therapeutic approaches and medications, and provides direction for the design of clinical research.

### 4.2 Investigating neuro-immune circuits with cutting-edge technologies

Identifying the neural circuits behind sympathetic storms and their downstream effects is essential for understanding disease mechanisms and developing targeted therapies. Advanced tools like optogenetics and chemogenetics enable precise modulation of specific neurons, providing insights into the sympathetic nervous system’s role in disease development (Veerakumar et al., 2022; Wengrowski et al., 2015). Furthermore, optogenetics has demonstrated potential for application in therapeutic exploration. A recent study introduced a high-performance optogenetic inhibition tool called HcKCR1-hs. This novel tool enables non-invasive transcranial light activation, effectively suppressing epileptiform discharges and seizure behaviors in mice (Duan et al., 2025; Govorunova et al., 2022). It also allows real-time regulation of hippocampal network function, providing experimental evidence for new strategies in epilepsy treatment (Andrews et al., 2024). This breakthrough highlights the promise of optogenetics in the functional modulation of neural circuits related to pathological conditions. Chemical genetics achieve reversible modulation of specific neurons through chemically induced receptor expression systems, which could also provide powerful means to analyze the neural circuits behind sympathetic storms (Gannot et al., 2024). Both optogenetics and chemogenetics not only help scientists gain a deeper understanding of the specific mechanisms underlying the role of the SNS in disease development but also lay a solid foundation for the design of targeted therapeutic strategies based on neural circuit modulation. These advancements suggest that combining neural circuit research with therapeutic strategy development represents a critical direction for future neuroscience investigations.

### 4.3 Interdisciplinary collaboration

Neurogenic pulmonary edema research is inherently interdisciplinary, so multidisciplinary collaboration is critical to advancing the field. Currently, there is insufficient collaboration between neuroscience, immunology, engineering, and biomedical technology, resulting in slow progress in research and applications. Although there have been initial collaborations between neuroscience and immunology in some areas, there is a lack of systematic integration. Neuroscience focuses on the pathophysiological changes following CNS injury, while immunology focuses more on the inflammatory response and its regulatory mechanisms. The combination of the two could shed more light on the pathogenesis of NPE, for example, by analyzing changes in the dynamics of inflammatory factors and their impact on pulmonary hemodynamics (Jin et al., 2021; Li R. et al., 2022). Future initiatives should promote collaborative research between neuroscience and immunology to thoroughly explore the “neuroimmune lung” axis. Integrating recent advances in molecular biology, engineering and medical imaging will help to advance dynamic biomarker monitoring and synchronized imaging molecular diagnostic techniques.

## 5 Conclusion

The framework constructed in this review has direct extensions beyond neurogenic pulmonary edema. Sympathetic storm and neuroimmune dysregulation are central to the pathogenesis of several other conditions, including Takotsubo cardiomyopathy, post-viral dysautonomia, severe COVID-19, autonomic dysfunction after traumatic brain injury, and other syndromes characterized by sympathetic hyperactivation and systemic inflammation. The spatiotemporal principles and therapeutic strategies outlined here, stage-specific sympathetic blockade, neuromodulation targeting the cholinergic anti-inflammatory pathway, and restoration of glymphatic clearance, may therefore have broader applicability. Future research should explore whether the neurotransmitter-immune-inflammatory model and its predictions translate to these related conditions.

Advances in precision medicine have introduced promising frameworks for addressing the challenges of treating NPE. With a deeper understanding of its pathological mechanisms and the application of multi-target approaches, clinical management is moving toward personalized diagnostics and tailored interventions. The integration of dynamic biomarkers, advanced imaging techniques, and innovations in genomics and biotechnology offers a transformative opportunity to develop cross-system, staged treatment strategies with improved therapeutic efficacy. Looking ahead, regulating key pathways such as the brain-lung axis and neuro-immune interactions will enable the design of more effective therapies, ultimately improving patient outcomes and quality of life. Furthermore, insights gained through NPE management can provide valuable guidance in diagnosing and treating other complex conditions influenced by systemic neural and immune interactions.
